# Community based multi-disease health screening as an opportunity for early detection of HIV cases and linking them to care

**DOI:** 10.1186/s12889-023-15948-6

**Published:** 2023-06-01

**Authors:** Christopher Z-Y. Abana, Dennis K. Kushitor, Theodore W. Asigbee, Prince K. Parbie, Koichi Ishikawa, Hiroshi Kiyono, Taketoshi Mizutani, Samuel Siaw, Sampson B. Ofori, Gifty Addo-Tetebo, Maclean R. D. Ansong, Marion Williams, Samuel Morton, George Danquah, Tetsuro Matano, William K. Ampofo, Evelyn Y. Bonney

**Affiliations:** 1 Virology Department, Noguchi Memorial Institute for Medical Research (NMIMR), University of Ghana, Accra, Ghana; 2 West African Center for Cell Biology of Infectious Pathogens (WACCBIP), University of Ghana, Accra, Ghana; 3 Department of Biochemistry, Cell and Molecular Biology, College of Basic and Applied Sciences, University of Ghana, Accra, Ghana; 4 Eastern Regional Hospital, Koforidua, Ghana; 5 AIDS Research Center, National Institute of Infectious Diseases (NIID), Tokyo, Japan; 6 Joint Research Center for Human Retrovirus Infection, Kumamoto University, Kumamoto, Japan; 7 Institute of Medical Sciences, The University of Tokyo, Tokyo, Japan; 8 Future Medicine Education and Research Organization, Institute for Global Prominent Research, Graduate School of Medicine, Chiba University, Chiba, Japan; 9 Allergy and Vaccines (cMAV), Department of Medicine, Chiba University-University of California San Diego Center for Mucosal Immunology, University of California San Diego, Chiba, USA

**Keywords:** Community-based, Multi-disease screening, Know your status, HIV

## Abstract

**Background:**

The 95-95-95 UNAIDS global strategy was adapted to end the AIDS epidemic by 2030. The target is based on the premise that early detection of HIV-infected persons and linking them to treatment regardless of their CD4 counts will lead to sustained viral suppression. HIV testing strategies to increase uptake of testing in Western and Central Africa remain inadequate. Hence, a high proportion of people living with HIV in this region do not know their status. This report describes the implementation of a community based multi-disease health screening (also known as “Know Your Status” -KYS), as part of basic science research, in a way that contributed to achieving public health goals.

**Methods:**

A community based multi-disease health screening was conducted in 7 communities within the Eastern region of Ghana between November 2017 and April 2018, to recruit and match HIV seronegative persons to HIV seropositive persons in a case-control HIV gut microbiota study. Health assessments included blood pressure, body mass index, blood sugar, Hepatitis B virus, syphilis, and HIV testing for those who consented. HIV seronegative participants who consented were consecutively enrolled in an ongoing HIV gut microbiota case-control study. Descriptive statistics (percentages) were used to analyze data.

**Results:**

Out of 738 people screened during the exercise, 700 consented to HIV testing and 23 (3%) were HIV positive. Hepatitis B virus infection was detected in 4% (33/738) and Syphilis in 2% (17/738). Co-infection of HIV and HBV was detected in 4 persons. The HIV prevalence of 3% found in these communities is higher than both the national prevalence of 1.7% and the Eastern Regional prevalence of 2.7 in 2018.

**Conclusion:**

Community based multi-disease health screening, such as the one undertaken in our study could be critical for identifying HIV infected persons from the community and linking them to care. In the case of HIV, it will greatly contribute to achieving the first two 95s and working towards ending AIDS by 2030.

## Introduction

Human Immunodeficiency Virus (HIV) continues to be a major global public health problem with an estimated 38.0 million people living with HIV (PLWH) with the greatest impact in Africa (25.7 million) [1]. In 2019, the estimated adult HIV prevalence for Ghana was 1.7% with an estimated HIV population of 316, 352 and 25, 955 adults and children respectively [2]. In the same year, Ghana’s Eastern Region recorded an HIV prevalence of 2.7% according to the annual HIV Sentinel Survey conducted by the National AIDS/STIs Control Programme [3].

Early detection, coupled with rapid linkage to care is particularly essential in mitigating the HIV/AIDS epidemic. However, the proportion of people living with HIV (PLWH) who know their status remains inadequate in Western and Central Africa populations [4]. As of 2017, only 48% of people living with HIV in Western and Central African knew their status [4]. Scaling-up antiretroviral therapy (ART) is crucial to the control of the HIV/AIDS pandemic [5]. Initiation of early ART does not only reduce morbidity and mortality but also reduces incidence rates of HIV, resulting in the concept of “Treatment as Prevention (TasP)” [6, 7]. To maximize the benefits of TasP, PLWH must be diagnosed early in the course of their HIV infection, link to care and remain adherent to ART to attain full virologic suppression [8]. However, due to poor health-seeking behaviour coupled with socio-cultural dynamics, most PLWH in Africa do not access health facility-based voluntary counselling and testing services resulting in delayed diagnosis.

In response to the 95-95-95 global strategy, by the Joint United Nations Programme on HIV/AIDS to accelerate efforts toward ending the AIDS epidemic by 2030 [9], the Ghana National AIDS/STI Control Programme (NACP) developed a 5-year road map to Locate, Test, Treat and Retain (L2TR) [10]. The road map focussed on lower-level health workers, volunteers, and other stakeholders but also included expanding community health screening, also known as HIV “know your status” (KYS) campaigns to scale up HIV testing in communities and timely ART initiation. The KYS campaign is an outreach program that was introduced in Ghana in 2007 following its success in other resource-limited settings to encourage the general population to know their HIV status [11]. This innovative approach to HIV testing which has been demonstrated to improve testing uptake [12, 13] is an important step in realizing the goal of diagnosing at least 95% of PLWH in the UNAIDS strategy to end the HIV epidemic [9].

Most large-scale community-based health screening campaigns are usually designed to create awareness of pertinent health issues. A few like the KYS goes a step further by linking participants to treatment and care services.

Here, we describe how we implemented a community based multi-disease health screening to recruit healthy controls in seven communities in an ongoing HIV Microbiome study [14] and in the process identified PLWH and linked them to care. Data on the HIV-Microbiome study has been published elsewhere [14]. The HIV-Microbiome study is under the project for Science and Technology Research Partnership for Sustainable Development Program (SATREPS). It is a collaborative project between the Noguchi Memorial Institute for Medical Research (NMIMR), Eastern Regional Hospital, Koforidua, Ghana Health Service (GHS), and Japanese partner institutions (National Institute of Infectious Diseases- NIID, Institute of Medical Sciences, the University of Tokyo).

## Methods

### Study design

**Fig. 1:**
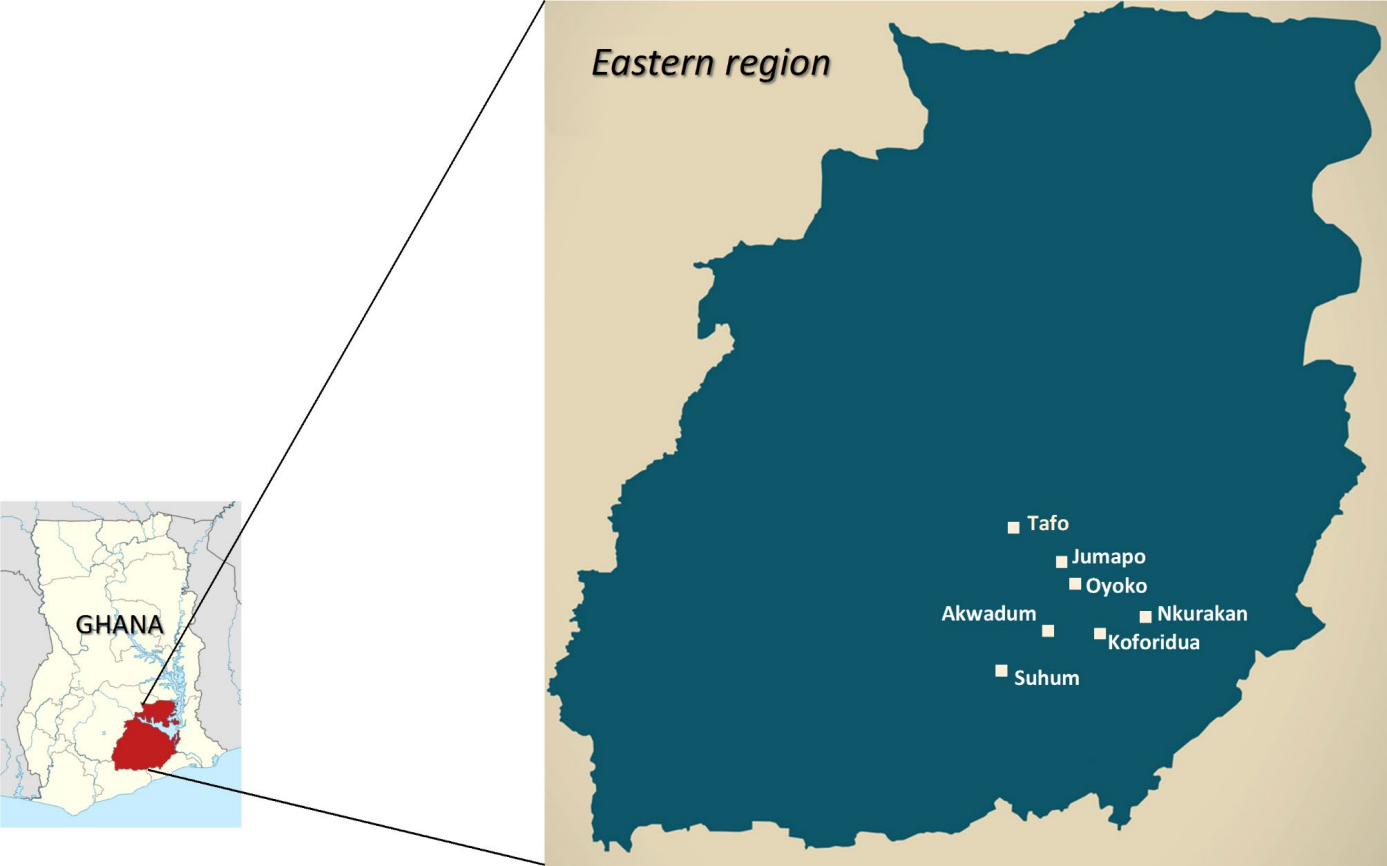
Map showing the 7 communities in eastern region of Ghana

This was a cross-sectional study conducted from November 2017 to April 2018 in 7 communities in the Eastern Region of Ghana. The regional hospital, located in Koforidua, serves as the main HIV and ART clinic and referral center in the region; with the responsibility of coordinating HIV/AIDS prevention and intervention programmes and providing care and support to PLWH in the region. Our case-control HIV Gut Microbiome Study recruited HIV seropositive persons from the regional hospital and needed seronegative persons from their resident communities to match. HIV screening was therefore conducted in these communities as part of a community-based multi disease screening to identify and recruit HIV seronegative persons. Participants were recruited from 7 communities in 4 districts of the Eastern Region where the majority of the HIV-positive persons seeking care at the Eastern Regional Hospital in Koforidua reside. The 7 communities were Koforidua, Oyoko, Akwadum, Jumapo (New Juaben District), Nkurakan (Yilo Krobo District), Tafo (East Akim District), Suhum (Suhum District) as shown in the map in Fig. 1.

### Study population

#### Enrolment of study participants from each community

In each selected community, information vans were used to announce the arrival of the health team in the community and to indicate the date, time and venue for the health screening exercise. A durbar (public reception) was organised in each community. During the durbar, the Regional HIV/AIDS Coordinator gave a welcome address and a talk on HIV. This talk covered the importance and benefits of knowing one’s HIV status, as well as checking for other infections and health indicators such as syphilis, Hepatitis B, obesity, and Diabetes. Participants were allowed to consent and undertake their choice of tests among all that was on offer: syphilis, hepatitis B, HIV and body mass index (BMI). Each participant then proceeded to the laboratory for their chosen test to be performed. Only consenting persons were tested for HIV. Participants were screened for syphilis and HIV using First Response Rapid HIV syphilis combo card test kit (Premier Medical Corporation Ltd, India). Hepatitis B screening was done using SD Bioline HBsAg (Standard Diagnostic Incorporated, Korea). The BMI was calculated using the Metra BYH01-BMI weight and height scale (Metra, UAE). Participants were counselled and presented with their results. All individuals with out-of-range health indicators were referred to local health facilities for further assessment and management.

HIV positive persons were referred to the Eastern Regional Hospital, Koforidua to be linked to care and initiated on antiretroviral therapy. Consent was sought from matched HIV seronegative persons to be consecutively enrolled in the study. Unmatched participants and those who did not consent were excluded from the study.

## Results

A total of 738 persons from the 7 communities participated in the health screening exercise with females constituting the highest number 68% (500/738). Over 70% had formal education, 48% were traders and over 50% were not married. Some participants (48%) have ever participated in a health screening exercise but only 30% and 12% have ever tested for HIV and HBV respectively (Table 1). A clear majority of the participants (71%) just wanted to know their health status.

The health screening exercise varied significantly between males and females across the communities (p < 0.001). Half (50%) of the participants had a normal BMI. There was a significant association between BMI and the communities; with the highest number of people with normal BMI coming from Koforidua township. Similarly, the reason for the health screening, educational status (p < 0.001), occupation (p < 0.001), and marital status (p < 0.001) also varied significantly across the 7 communities. However, there was no significant association between testing positive for syphilis and gender. Furthermore, there was no significant association between the various indicators (Syphilis, HIV, and HBV) and age groups.

All 738 persons recruited consented to syphilis and HBV testing. However, 38 of them did not consent to be tested for HIV. Of the 700 participants that tested for HIV, 23 (3%) were positive while 2% (17/738) were positive for syphilis and 4% (33/738) for HBV infection (Table 2). Co-infection of HIV and HBV was detected in 4 participants. However, there was no co-infection of syphilis with either HIV or HBV. Tafo and Suhum communities recorded the highest proportion (6.8% and 6.0% respectively) of HIV-infected individuals (Table 2). A higher proportion of males were infected with HBV (Males = 13/238, 6%; Females = 20/500, 4%) and syphilis (Male = 9/238, 4%; Females = 8/500, 2%). In contrast, the proportion of males infected with HIV (6/226, 3%) was lower compared to females (17/474, 4%). The majority of the participants were older than 40 years (441/738) (Table 3). However, the proportion of participants infected with syphilis, HBV, and HIV was higher among individuals in the age group less than 40 years. The highest proportion of syphilis (4%) was detected in the 31–35 year age group. The highest proportion (7%) of HBV was detected in the 36–40 year age group. Likewise, the highest proportion (7%) of HIV was detected in the 36–40 year age group.

**Table 1:** Demographics of participants in the community health screening

		Community									
		Koforidua	Suhum	Tafo	Nkurakan	Oyoko	Akwadum	Jumapo	Total		P-value
Total Recruited	Male	100	20	72	32	8	3	3	238	32%	< 0.001
	Female	141	67	99	178	5	6	4	500	68%	
	Total	241	87	171	210	13	9	7	738		
	%	33%	12%	23%	28%	2%	1%	1%			
BMI	Normal	128	33	86	111	2	3	3	366	50%	< 0.001
	Underweight	31	12	18	16	2	1	2	82	11%	
	Overweight	55	2	59	54	4	3	1	178	24%	
	Obese	27	40	8	29	5	2	1	112	15%	
Educational Status	Educated	201	81	111	157	7	5	3	565	77%	< 0.001
	Not Educated	40	6	60	53	6	4	4	173	23%	
Occupation	Trader	90	40	94	126	4	2	1	357	48%	< 0.001
	Student	46	13	36	21	3	3	2	124	17%	
	Teacher	45	18	18	22	1	0	2	106	14%	
	Farmer	35	9	5	24	4	3	4	84	11%	
	Others	23	7	18	17	1	1	0	67	9%	
Marital Status	Married	117	33	50	104	6	3	4	317	43%	0.001
	Not Married	124	54	121	106	7	6	3	421	57%	
Ever participated in a community health screening	Yes	110	38	129	68	2	3	1	351	48%	< 0.001
	No	131	49	42	142	11	6	6	387	52%	
Reason for the health screening	To know my health status	135	52	132	185	10	5	4	523	71%	< 0.001
	To test for HIV	70	23	25	15	3	3	2	141	19%	
	To test for HepBsAg	36	12	14	10	0	1	1	74	10%	
Ever Tested for HIV	Yes	129	32	22	31	1	2	1	218	30%	< 0.001
	No	112	55	149	179	12	7	6	520	70%	
Ever Tested for HepBsAg	Yes	45	10	20	15	0	1	0	91	12%	0.009
	No	196	77	151	195	13	8	7	647	88%	

**Table 2:** Summary of STI test results

Indicators			Community										
			Koforidua	Suhum	Tafo	Nkurakan	Oyoko	Akwadum	Jumapo	Total (%)		P-value	
Total Screened (N = 738)		Male	100	20	72	32	8	3	3	238	(32%)	< 0.001	
		Female	141	67	99	178	5	6	4	500	(68%)		
		Total	241	87	171	210	13	9	7	738			
		%	33%	12%	23%	28%	2%	1%	1%				
Syphilis (VDRL) Positive (N = 738)		Male	7	1	1	0	0	0	0	9	17 (2%)	0.52	
		Female	2	0	3	3	0	0	0	8			
		Total	9	1	4	3	0	0	0	17			
		%	3.7%	1.1%	2.3%	1.4%	0.0%	0.0%	0.0%				
HBsAg (N = 738)		Male	8	2	1	0	2	0	0	13	33 (4%)	0.190	
		Female	8	2	3	6	1	0	0	20			
		Total	16	4	4	6	3	0	0	33			
		%	6.6%	4.6%	2.3%	2.9%	23.1%	0.0%	0.0%				
HIV	Total tested (N = 700)	Male	95	19	68	30	8	3	3	226	(32%)	< 0.001	
		Female	134	64	94	169	5	5	3	474	(68%)		
		Total	229	83	162	199	13	8	6	700			
		%	32.7%	11.9%	23.1%	28.4%	1.9%	1.1%	0.9%				
	Positive	Male	1	1	4	0	0	0	0	6	23 (3%)	0.624	
		Female	3	4	7	3	0	0	0	17			
		Total	4	5	11	3	0	0	0	23			
		%	1.7%	6.0%	6.8%	1.5%	0.0%	0.0%	0.0%				

**Table 3:** Age group of persons who participated in the community health screening

		Age groups (years)											
		15–20	21–25	26–30	31–35	36–40	41–45	46–50	> 50	Total		P-value	
HIV	Tested	10	59	68	69	77	66	83	268	700			
	Positive	0	3	2	2	5	2	2	7	23		0.769	
	%	0.0%	5.1%	2.9%	2.9%	6.5%	3.0%	2.4%	2.6%	3%			
Syphilis	Tested	11	60	72	73	81	70	88	283	738			
	Positive	0	1	1	3	1	1	1	9	17		0.798	
	%	0.0%	1.7%	1.4%	4.1%	1.2%	1.4%	1.1%	3.2%	2%			
HBV	Tested	11	60	72	73	81	70	88	283	738			
	Positive	0	4	5	1	6	2	3	12	33		0.517	
	%	0.0%	6.7%	6.9%	1.4%	7.4%	2.9%	3.4%	4.2%	4%			

## Discussion

Community-based multi-disease screening, such as the one undertaken in our study could be critical for identifying HIV infected persons from the community and linking them to care. This study will greatly contribute to achieving the first two 95s towards ending the AIDS pandemic by 2030. Using this approach, we were able to recruit HIV seronegative persons as a control cohort for an ongoing study on HIV and Gut Microbiota [14]. Participants identified to be infected with HIV were referred for counselling and linked to care at the community health facilities. Of the total number of individuals recruited, 52% were participating in a community health screening such as KYS for the first time. We observed that HIV/STI testing uptake was very low in our study population; 30% and 12% of participants had ever tested for HIV and HBV respectively. The lower patronage could be attributed to stigma and discrimination. It was shown that a major barrier to voluntary testing and counselling in sub-Saharan Africa is the stigma and discrimination associated with HIV/AIDS [15–17]. In this study, pre-test screening revealed that the majority (71%) of the participants wanted to know their general health status but not HIV and HBV status. Only 11% and 10% wanted to know their HIV and HBV status respectively. However, through comprehensive education, counseling, and integration of HIV testing with a general health screening in our KYS model, we were able to consent all participants for HBV testing and 95% (700/738) for HIV testing. This indicates that through appropriate education, counselling, and sensitization methods, the impact of stigmatization and discrimination on HIV testing uptake could be significantly reduced as recommended elsewhere [16]. Also, educational level is reported as a predictor of uptake of HIV testing [17, 18]. In this study we observed that 77% of participants have had formal education, thus contributing to the positive response of consenting to HIV-STI testing.

We observed that the majority (68%) of participants in the study were females (Table 1). This could be because females in Ghana are known to exhibit a more favorable health-seeking behavior compared to the males [17, 18]. However, it is also worth noting that females constitute a majority (51%) of the population of the Eastern Region [19]. Thus, the higher proportion of female participants in this study may not be entirely attributable to better health seeking behavior in women.

Though the Eastern Region has a youthful population structure [19], we observed that more participants (59%) in this study were older than 40 years, while there was low patronage from the youth (Table 3). This is consistent with other studies that reported that stigma and fear associated with knowing one’s health status, especially HIV, discourage the youth from participating in KYS campaigns [15–18, 20–22]. However, the proportion of participants infected with syphilis, HBV, and HIV was higher among individuals in age groups less than 40 years. Particularly, individuals in the 36–40 years age group recorded the highest proportion of HIV (7%) and HBV (7%) infections (Table 3). All 4 cases of HIV-HBV co-infection were recorded in the same age group. We did not detect co-infections with syphilis.

Women are disproportionately affected by the HIV/AIDS epidemic in sub-Saharan Africa [23]. Consistently, we recorded a higher proportion of females (17/474, 3.6%) infected with HIV compared to males (6/226, 2.7%) (Table 2). In contrast, a higher proportion of males were infected with HBV (13/238, 5.5%) and Syphilis (9/238, 3.8%) than females. The overall HIV prevalence (3%) found in this study is higher than both the national prevalence of 1.7% [24] and the Eastern Regional prevalence of 2.7% in 2017 [25]. Routine community based multi disease health screening campaign models such as the one reported in this study and other studies [26–28] could be a spring board to inform prevention and control strategies for HIV/AIDS and other STIs.

Community based testing and counselling is part of the HIV testing approaches used in Ghana to improve achievement of the first 95 of the UNAIDS target goals for 2030 [10, 24]. However, uptake of HIV testing remains low. In this study we demonstrated that integrating HIV testing into a general health screening in communities improves HIV testing uptake. Adapting a model such as ours could contribute to improving the first two 95s of the UNAIDS 95 95 95 target by 2030[9].

## Data Availability

All data generated or analysed during this study are included in this published article.
